# Research on the influencing factors of Chinese agricultural brand competitiveness based on DEMATEL-ISM

**DOI:** 10.1038/s41598-024-62068-1

**Published:** 2024-05-18

**Authors:** Huanchen Tang, Xiaodong Liu, Meiyu Li

**Affiliations:** 1 College of Fashion and Art Design, Donghua University, Shanghai, 200051 China; 2 Xianda College of Economics and Humanities, Shanghai International Studies University, Shanghai, 200216 China

**Keywords:** Agricultural products, Brand competitiveness, DEMATEL-ISM, Yangtze River Delta region, Environmental economics, Socioeconomic scenarios

## Abstract

Agricultural products are pivotal to the national economy, and a comprehensive analysis of brand competitiveness significantly contributes to the support of agricultural structural adjustment and modernization. Focusing on the Yangtze River Delta region of China, this study develops an evaluation index system encompassing four dimensions: core brand competitiveness, brand management, market competitiveness, and innovation in branding. Utilizing a DEMATEL-ISM model, this research elucidates the intrinsic relationships among factors that influence brand competitiveness, resulting in a four-tier hierarchical model. The analysis delineates key factors at superficial, intermediate, and profound levels that influence brand competitiveness. Notably, regional production bases, along with innovations in brand technology and systems, emerge as profound influencers. Drawing on these findings, the study recommends strategies to enhance production foundations, accurately define development trajectories, spearhead technological innovation to foster collective reform efforts, and advocate for institutional advancements to bolster healthy brand growth.

## Introduction

**Figure 1:**
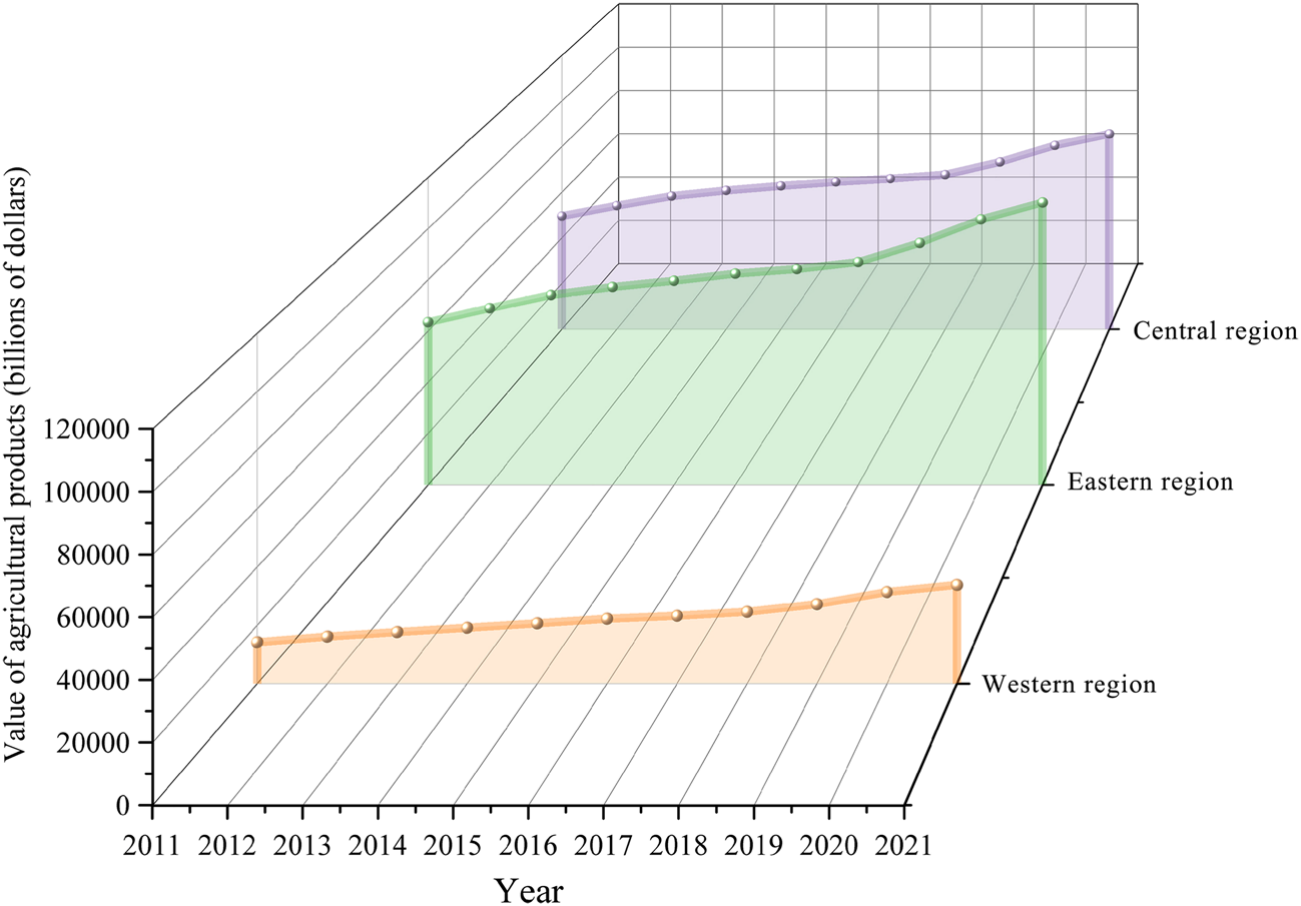
Trends in the value of agricultural products in China's eastern, central and western regions, 2011–2021.

**Figure 2:**
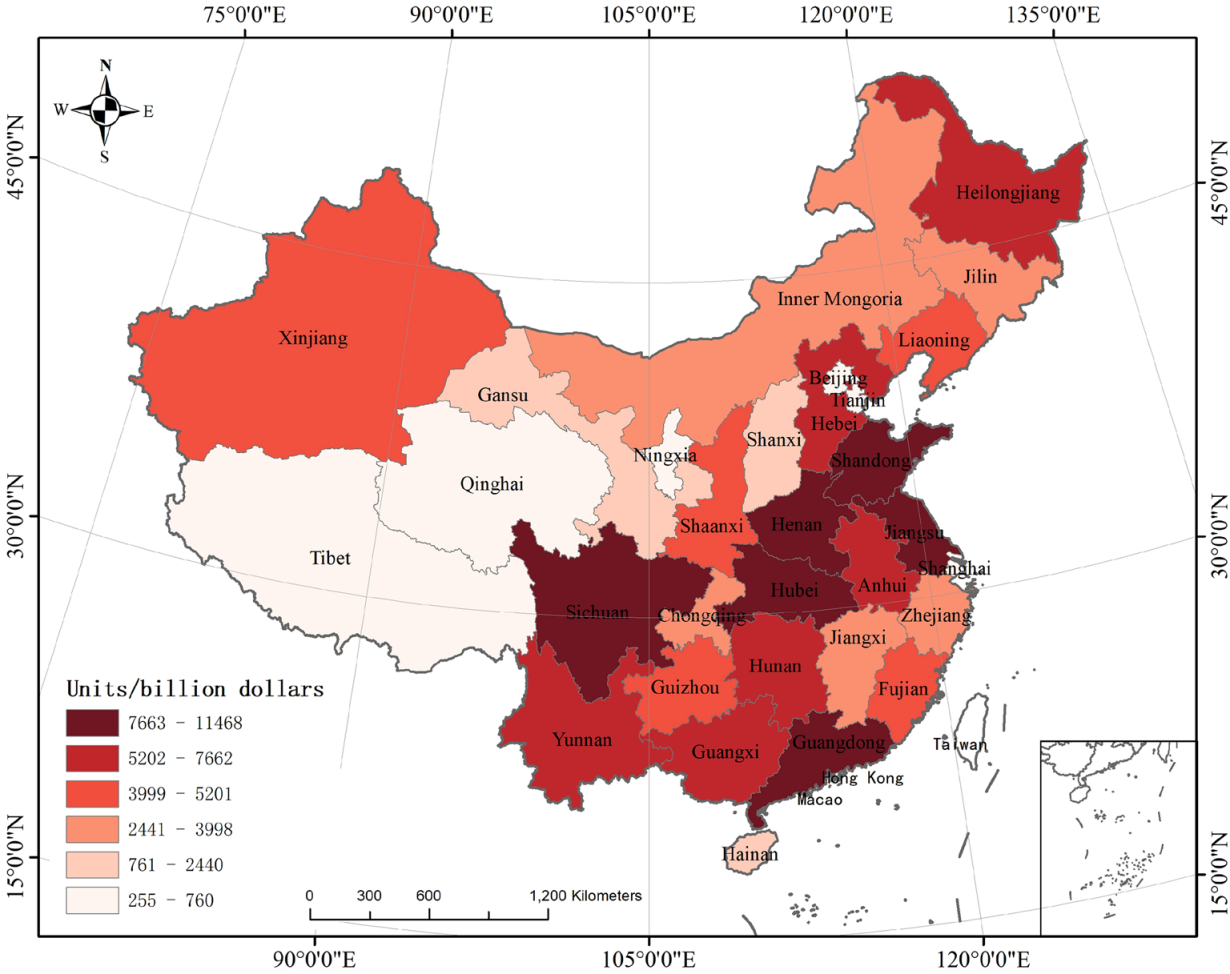
Heat Map of China's gross output value of agricultural products, 2021.

As a principal agricultural producer, China's agricultural product competitiveness significantly influences the country's agricultural progress^1^. Over the past four decades, due to reform and opening-up policies, China has seen substantial improvements in agricultural productivity. However, its agricultural development remains less advanced compared to developed nations^2^. Agricultural product branding, a critical element of the rural revitalization strategy, plays a pivotal role in fostering agricultural advancement and transformation, enhancing the quality and efficiency of products, increasing farmers' incomes, and broadening consumer demand^3^. Since 2007, the Chinese government has repeatedly underscored the significance of agricultural product branding, advocating for robust initiatives to promote the establishment of regional public brands, corporate brands, and enhance branding efforts, and to implement the 'three products and one standard' (variety optimization, quality improvement, brand creation, and standardized production) strategy to facilitate higher levels of green agricultural development^4^. The 19th National Congress of the Communist Party of China in 2017 highlighted the critical issues of agriculture, rural areas, and farmers as fundamental to national welfare, placing the resolution of these 'three rural issues' at the forefront of its agenda^5^. Enhancing agricultural product brand competitiveness through the rural revitalization strategy is vital for market expansion, income increase, brand loyalty enhancement, international trade promotion, industrial chain optimization, and rural industry restructuring, establishing it as an essential component of the strategy. The No. 1 central document of 2017 advocated for the development of regional public brands and encouraged local governments to develop regional specialty brands supported by leading enterprises and industry associations^6^. The "National Rural Revitalization Strategy Plan (2018–2022)" explicitly called for a faster development of these brands to boost market competitiveness^7^. Subsequent documents in 2020^8^, 2021, and 2022 emphasized strengthening brand building and creating distinctive, 'small yet beautiful' specialty agricultural brands, reinforcing China's commitment to branded agricultural development^9^. The ongoing refinement of the agricultural branding system, marked by significant enhancements in brand benefits and more diversified distribution channels, underscores its strategic significance in China's agricultural advancement. Currently, China's agricultural product market confronts several significant challenges, including variable product quality, redundant branding for single products, low brand recognition, and inadequate branding efforts. The uneven and insufficient development of agricultural product branding is increasingly evident, becoming a major constraint on the rising consumer demand^10^. The third national agricultural census in China yielded trend charts of agricultural output values across the eastern, central, and western regions from 2011 to 2021 (Fig. 1) and for 2021 specifically (Fig. 2)^11^. According to Fig. 1, agricultural output is predominantly higher in the eastern regions, while it is markedly lower in the central and western regions. Figure 2 illustrates that cities with substantial agricultural output predominantly cluster in the Yangtze River Delta, Pearl River Delta, and Bohai Economic Rim—areas known for their strategic economic importance and robust agricultural bases^12^. The "China Agricultural Product Brand Development Research Report" reveals that Chinese agricultural product brands are notably deficient in quality, quantity, scale, and efficiency, complicating the sale of these products and making brand building particularly challenging^13^. National policies have consistently advocated for enhancing agriculture through quality and branding, promoting environmentally sustainable, high-quality, specialized, and branded agricultural development^14^. Specifically, in 2020, the government escalated its expectations, focusing on establishing renowned agricultural product brands and augmenting the supply of premium, eco-friendly agricultural products^15^. What factors influence the competitiveness of agricultural product brands? How can the competitiveness of agricultural product brands be enhanced? Through what channels do these factors influence the competitiveness of agricultural product brands? Answering these questions is also the purpose and significance of this study.

For agricultural product producers and operators, the ability to objectively assess the competitiveness of their brands is vital. Such assessments enable a comprehensive understanding of their brand's current status and challenges, informing strategic planning for brand development. To this end, this study employs the DEMATEL (Decision-Making Trial and Evaluation Laboratory) structural model to examine the factors influencing the competitiveness of agricultural product brands in the Yangtze River Delta region of China. Building on this analysis, the paper proposes a scientifically sound and rational evaluation system for assessing agricultural brand competitiveness, thereby providing theoretical and practical insights for enhancing China's agricultural branding, boosting brand competitiveness, and supporting rural revitalization. This paper's innovations are twofold: first, it broadens the research perspective by focusing on brand competitiveness; second, it utilizes the DEMATEL-ISM model not only to analyze the determinants of brand competitiveness but also to uncover the underlying mechanisms of these factors.

## Literature review

### Brand competitiveness of agricultural products

Agricultural product branding significantly enhances farmers' incomes and elevates the quality and efficiency of agricultural practices. This branding is fundamental to achieving greater market influence, increased market share, and enhanced added value for branded products over unbranded counterparts^16^. Serving as an extension of branding within the agricultural sector, it plays an essential role in facilitating communication between agricultural operators and consumers. Moreover, it boosts consumer recognition of agricultural enterprises, as well as the origin and quality of their products^17^, simultaneously highlighting the company’s reputation and its commitment to consumers.

Branding fundamentally involves an implicit contractual relationship between businesses and consumers, where businesses commit to providing high-quality products or services, and consumers reciprocate by paying a "brand premium," thus establishing a balanced market exchange model. This deepened relationship underpins brand competitiveness^18^. Agricultural product brand competitiveness is defined by a company's capacity to integrate both internal and external resources while considering consumer psychology. Effective branding strategies—including positioning, communication, operation, and management—help cultivate a favorable brand image, enhance consumer recognition, stimulate purchase behavior, and foster brand loyalty^19^. Additionally, Abimbola’s research suggests that the competitiveness of agricultural product brands is intricately linked to commitments to social responsibility and sustainable development^20^.

The millennia-old agricultural civilization of China has created substantial historical wealth. However, due to the smallholder economic structure, cultural traditions, and production practices, Chinese agricultural practitioners often exhibit weak brand awareness, and the development of agricultural product branding is relatively underdeveloped. This shortfall in strong, sustainable competitive brands significantly limits the enhancement of China's agricultural competitiveness^10^. Agricultural products typically feature inherent quality opacity, resulting in informational asymmetry between buyers and sellers and complicating consumers' ability to discern product quality accurately^21^. Consequently, branding, which functions both as a symbol and a quality assurance, has become an essential, competitive necessity in agriculture. As the commercialization of agricultural products advances, domestic brand competition intensifies, compounded by increasing pressure from imported agricultural products^22^. Without robust core competitiveness, Chinese agricultural brands may find it difficult to maintain market presence. Moreover, in an era characterized by rapid technological advancements, faster product iterations, and diversifying communication methods, sustaining a competitive advantage through branding poses growing challenges.

### Factors affecting the competitiveness of agricultural brands

The foundational studies on brand competitiveness date back to the 1950s when Levy identified that brands significantly enhance consumer advantages in market competition^23^. Following this, scholarly research systematically explored brand competitiveness theory from diverse perspectives. For instance, Motamenti introduced a global asset model that evaluates brand competitiveness using customer potential, competitive potential, and global potential^24^. Tao Cai and colleagues developed a brand evaluation index system, considering brand value and focusing on six dimensions: positioning, personality, innovation, culture, communication, and customer engagement^25^. From a marketing and strategic standpoint, Suraksha Gupta and associates determined that brand differentiation positively impacts brand competitiveness^26^^.^ Yishu Liu and collaborators examined the relationship between the agglomeration effect of agricultural products and brand competitiveness, concluding that agricultural industry clusters enhance the value of green brands, boost competitive advantages, and augment overall brand competitiveness through collaborative efforts among agricultural research bodies, activities, service organizations, enterprises, and administrative departments^27^. Yaqi Jin et al. highlighted that market environment, regulations, consumer demand, and technological innovation are critical determinants of agricultural product brand competitiveness^28^. Anselmsson pointed out the importance of supply chain management, marketing strategies, and brand reputation in influencing brand competitiveness^29^. Additionally, Abimbola and others emphasized that agricultural product brand competitiveness encompasses brand image, product quality, market position, and consumer perception^20^.

### Literature review

In summary, scholars both domestically and internationally have conducted extensive research on brand competitiveness assessment. Studies specifically addressing agricultural product brands have mainly focused on qualitative analyses of definitions, connotations, and enhancement strategies for brand competitiveness. However, in developing brand evaluation systems, previous research has largely emphasized the intrinsic brand development capabilities of companies and their financial and market performance^30^, while often overlooking the influence of external factors such as competitors and the competitive environment. A comprehensive analysis based on existing literature reveals that factors influencing agricultural product brand competitiveness include industry competition factors (e.g., organizational scale and industry competitiveness)^31^, enterprise characteristics (e.g., information, culture, technology, human resources)^32^, and inherent brand factors (e.g., reputation and awareness)^26^. On a micro level, elements such as technology, design, positioning, marketing, and service within agricultural enterprises can directly or indirectly impact brand competitiveness^20^.

The competitiveness of agricultural product brands exhibits unique characteristics and is influenced by a variety of factors, thus requiring thorough investigation from both macroscopic and microscopic viewpoints that encompass the brands' intrinsic elements and the external environment. Furthermore, there is a notable deficiency in existing research regarding in-depth analysis specific to certain regions or agricultural products, coupled with a scant examination of how emerging technologies can boost the competitiveness of these brands. Consequently, this study, which builds upon previous theoretical frameworks, concentrates on the competitiveness of agricultural product brands in China's Yangtze River Delta. It develops an empirical model to explore the factors affecting brand competitiveness and investigates the direct and indirect relationships among these factors, thereby providing a more scientifically grounded basis for enhancing brand competitiveness.

## Overview of the study area and extraction of influencing factors

### Overview of the study area

**Figure 3:**
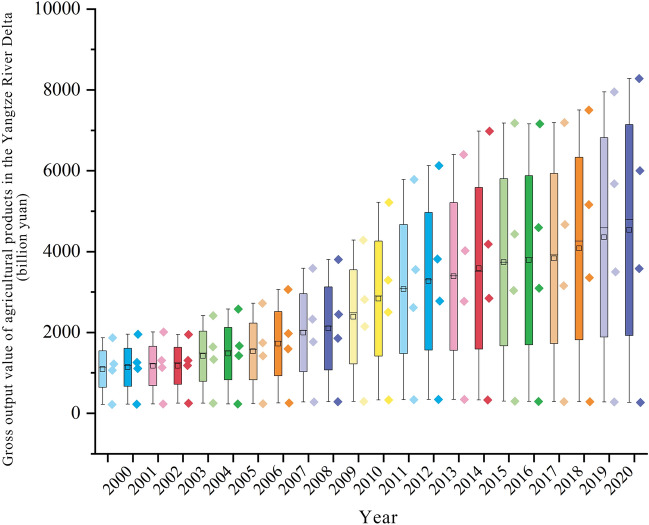
Boxplot of gross value of agricultural products in Yangtze River Delta.

The Yangtze River Delta, a pivotal nexus for the Belt and Road Initiative and the Yangtze River Economic Belt, includes Jiangsu, Zhejiang, Shanghai, and Anhui. Renowned as one of China's most open, innovative, and economically robust regions, it plays a significant role in the country's market dynamics^33^. Throughout the integration process, the region's agricultural product market has demonstrated significant potential, becoming an integral part of the broader market integration in the area. Analysis of data reveals that of China's top 100 agricultural enterprises, 22 are located in the Yangtze River Delta, contributing to approximately 15% of the total revenue and 19% of the market transactions^34^. Figure 3 shows that from 2000 to 2021, the overall output value of agricultural products—divided into crops, forestry, livestock, and fisheries—has consistently risen^35^.

The Yangtze River Delta region, a highly developed economic area in China, boasts a notable level of agricultural modernization and leads in financial innovation and reforms nationwide^36^. Despite these advancements, the region encounters challenges such as financial imbalances, suboptimal market structures, and inefficient financial resource allocation^37^. These challenges impede the development of a comprehensive agricultural industry chain, thus preventing the region from satisfying the national strategic imperatives for regional integration. Additionally, the Yangtze River Delta, a key economic hub in China, has experienced rapid enhancements in living standards, which in turn has spurred a strong demand for agricultural products. Consequently, analyzing the competitiveness of agricultural product brands in the Yangtze River Delta is of paramount importance.

### Influence factor extraction and interpretation

Initially, the search in the CNKI database employed "agricultural products" and "brand competitiveness" as primary keywords, restricting document types to "CSSCI", "PKU Core", "CSCD", and "AMI". Similarly, in the Web of Science, the query TS = (agricultural products* AND brand competitiveness) was utilized. Redundant and irrelevant studies were systematically excluded, culminating in the selection of 35 papers suitable for detailed review and analysis of evaluative factors.

Subsequently, integrating literature review findings with expert interviews, twelve determinants of agricultural product brand competitiveness were pinpointed and designated S1 through S12. These were organized into four categories: foundational, management, market, and innovative competitiveness, as illustrated in Table 1.

**Table 1:** Composition table of factors influencing the competitiveness of agricultural brands.

Composition of indicators	Indicator name	Indicator number	Indicator Factor Theory Description and Interpretation
Competitiveness of agricultural product brand base	Brand Culture	S_1_	An ecosystem for the innovative development of brands that can be integrated with quality enhancement systems and cognitive communication systems to showcase the excellence of local traditional cultures^25^
	Brand product quality	S_2_	Refers to aspects related to physical attributes such as function, safety, nutrients, taste, color, etc.^26^
	Regional production base	S_3_	Refers to the geographical environment, soil quality, temperature and humidity, sunshine and other natural conditions in the area where agricultural products are grown^27^
Competitiveness of agricultural brand management	Brand service level	S_4_	Refers to the service process of goods or services as a medium, in the economic activities to meet the psychological needs of consumers a unique form of brand expression^28^
	Brand Marketing Capabilities	S_5_	It refers to the comprehensive ability of an enterprise in the process of brand operation such as brand positioning, brand communication, brand extension and brand maintenance^29^
	Brand Image	S_6_	A brand has a set of symbols and image characteristics (e.g., shape, size, color, and other external image factors) that create a favorable effect on consumers^30^
Market competitiveness of agricultural brands	Brand awareness	S_7_	is the degree of awareness of an agricultural product brand among consumers, which reflects the number or proportion of consumers as a whole who are aware of the brand, and thus the scope or breadth of the product brand's influence in the marketplace^31^
	Brand Market Positioning	S_8_	It is the distinctive mark of the brand among similar competitive brands, the most core component of brand management elements, and also the essence of the brand^32^
	Market share	S_9_	It refers to the market share of the agricultural product brand in the domestic and international markets, the degree of popularity among consumers, and the percentage of sales volume (or sales) of the product among similar products in the market^33^
Competitiveness of agricultural brand innovation	Brand Technology Innovation	S_10_	It refers to the improvement of agricultural products through the application of relevant scientific and technological knowledge and cultivation experience in the selection of good seeds, cultivation technology, harvesting technology, packaging design, processing technology, storage technology, etc.^34^
	Brand System Innovation	S_11_	It refers to the planning and execution of brand strategy, which includes incentives provided by the country, region, industry, etc. for brand development^35^
	Brand Culture Innovation	S_12_	It refers to products with unique cultural connotations, which can be effectively combined with relevant agricultural cultural festivals, mainly including the comparative advantages of brand culture and the input of brand culture construction efforts^36^

Moreover, to improve the precision and efficacy of the factor analysis, following Northcutt et al.'s^38^ guidelines, a panel comprising 12 to 20 decision-making experts was assembled. This approach has been empirically confirmed as effective in further studies. The paper synthesized insights from discussions with 23 specialists and officials from agricultural management sectors, who assessed the relevance of each factor on a scale from no impact (0 points) to very significant impact (4 points).

Ultimately, the validation of content was achieved. The research conclusively mapped out the factors affecting brand competitiveness in agriculture across the four identified dimensions, detailed in Table 1.

## Research methodology and process

### Overall process

**Figure 4:**
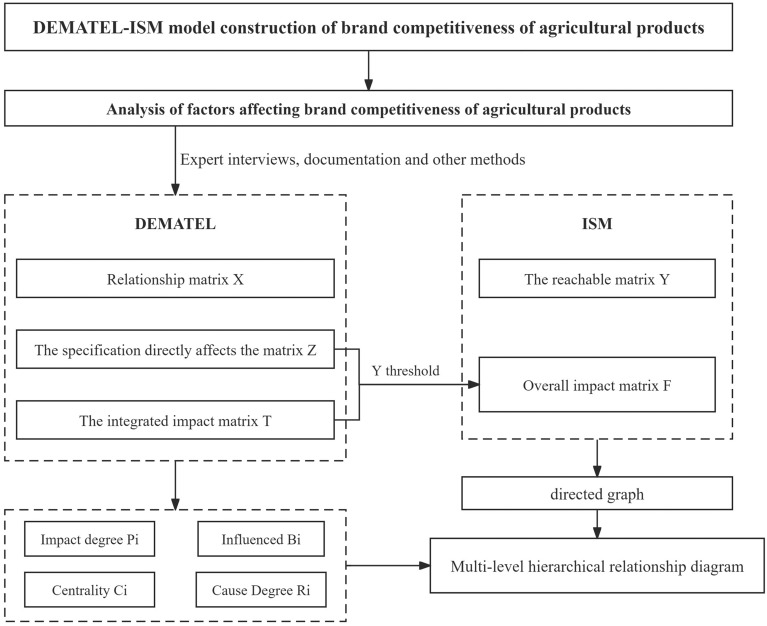
DEMATEL-ISM operation flowchart.

DEMATEL is a methodology devised by BOTTELLE Laboratories, employing graph theory, matrix tools, and expert knowledge to identify and analyze factors, thereby facilitating the resolution of complex real-world problems. This method operates without assumptions, enabling the exploration of logical relationships among elements and the determination of their importance and strategic status within the system^39^. Nonetheless, it lacks the ability to intuitively represent the interactions between internal factors. To overcome these shortcomings, this paper introduces the ISM (Interpretive Structure Modeling) technique. ISM further refines the comprehensive impact matrix using SPSS software, forming a multi-layered, sequential structure model that enhances the understanding of agricultural product brand competitiveness. This compensates for the deficiencies identified in the DEMATEL method^40^. By integrating these methods, a synergistic benefit is achieved, allowing for the intuitive visualization of relationships among factors and assisting in the selection of pivotal factors. This integration enhances the rigor and accuracy of the strategic recommendations and pathway analysis presented in this paper. Detailed procedural steps are illustrated in Fig. 4.

### DEMATEL method

#### Determine the influencing factors and establish the relationship matrix X

Identify the set of influencing factors $S = \left\{ {S_{i} \left| i \right. = 1,2, \ldots ,n} \right\}$, with Sij denoting the degree of influence of factor Si on Sj. For identical indices (i = j), S_ij_ is set to 0. Construct the relationship matrix X, defined as $X=\left[{S}_{ij}\right]n\times n$.

#### Calculation of the integrated impact matrix

Normalize using the row and maximum value methods, then compute the relationship matrix X employing Formula (1) to derive the standardized direct impact matrix Z. In this formula, the indices i and j range from 1 to n.

1 $$Z = \frac{X}{{_{{1 \le i \le n}}^{{max}} \sum\limits_{{j = 1}}^{n} {X_{{ij}} } }}$$

Building on the standardized direct impact matrix Z, this study elaborates the indirect relationships among factors by constructing the comprehensive impact matrix T, as specified in Formula (2). In this formula, 'A' denotes the identity matrix, with indices i and j ranging from 1 to n.

2 $$T=Z{\left(A-Z\right)}^{-1}$$

#### Calculating degree of centrality and degree of cause

The calculation for the degree of influence (P_i_) is given in Formula (3). In the formula: P_i_ represents the comprehensive impact value of factor i on other factors, with higher values indicating greater influence.

3 $${P}_{i}={\sum }_{j=1}^{n}{t}_{ij}$$

The calculation for the degree of being influenced (B_i_) is given in Formula (4). In the formula: B_i_ represents the comprehensive impact value that other factors have on factor i, with higher values indicating greater influence.

4 $${B}_{i}={\sum }_{j=1}^{n}{t}_{ij}$$

The calculation for centrality (C_i_) is given in Formula (5). In the formula: C_i_ indicates the level of importance of factor i, with higher values indicating greater importance.

5 $${C}_{i}={P}_{i}+{B}_{i}$$

The calculation for causality (R_i_) is given in Formula (6). In the formula: R_i_ represents the impact of factor i on other factors. If R_i_ > 0, it is a causal factor, influencing other elements more; if R_i_ < 0, it is a resultant factor, meaning it is more influenced by other factors.

6 $${R}_{i}={P}_{i}-{B}_{i}$$

#### Create centrality and causality diagrams

In this model, the horizontal axis denotes the centrality value P_i_(10)B_i_, and the vertical axis denotes the causality value P_i_—B_i_. The first quadrant, showing high _i_C and _i_R values, suggests that the factor is significant and causal. The second quadrant, with low _i_C and high _i_R values, indicates a less significant but still causal factor. The third quadrant, exhibiting low _i_C and R_i_ values, marks the factor as less significant and resultant. Conversely, the fourth quadrant, featuring high _i_C and low _i_R values, signifies that the factor is significant and resultant^39^.

### ISM method

#### Calculate the overall impact matrix F that can be used for ISM calculations

The specific calculation of the impact matrix F is shown in Formula (7). In the formula: i, j = 1, 2, …, n.

7 $$F=Z+T$$

#### Calculate the normalized reachability matrix Y

$Y=\left[{\gamma }_{\text{ij}}\right]n\times n$, where a threshold is introduced (the threshold $\gamma$ can be obtained by calculating the average value of all items in the comprehensive impact matrix T). If F_ij_ ≥ $\gamma$, then ${\gamma }_{\text{ij}}=1$, indicating that there is a connection path between elements; if F_ij_ < $\gamma$, then ${\gamma }_{\text{ij}}=0$, indicating that there is no connection path between elements.

#### Influence factor hierarchy and directed graph construction

Formulas (8) through (10) are utilized to derive the antecedent factor E(F_i_) and the reachable set G(F_i_), followed by the computation of their intersection, denoted as H(F_i_). If E(F_i_) equals H(F_i_), F_i_ is identified as the top-level factor, prompting its removal. This iterative process continues until all factors have been eliminated.

8 $$E\left({F}_{i}\right)=\left\{{F}_{i}\left|{F}_{j}\in F,{m}_{ij}=1\right.\right\}$$

9 $$G\left({F}_{i}\right)=\left\{{F}_{i}\left|{F}_{j}\in F,{m}_{ij}=1\right.\right\}$$

10 $$H\left({F}_{i}\right)=\left\{{F}_{j}\in F\left|E\left({F}_{i}\right)\bigcap G\left({F}_{i}\right)=E\left({F}_{i}\right)\right.\right\}$$

Finally, according to the hierarchical treatment, a directed graph and a schematic diagram of the hierarchical relationships between the influencing factors are created.

## Analysis of factors affecting brand competitiveness of agricultural products

### Calculation based on DEMATEL method

#### Establish the relationship matrix X

A five-level scoring method is employed to ascertain the magnitude of the factors' influence. In this approach, 0 denotes no influence of S_i_ on S_j_, while the influence level progresses incrementally from 1 to 4, with 4 denoting a highly significant influence. Twenty-three experts specializing in agricultural products and brand innovation, either through research or extensive expertise, were solicited to assign binary scores to the qualitative indicators delineated in this study. Subsequently, the relationship matrix X is formulated and presented in Table 2.

**Table 2:** Direct impact matrix X.

	S_1_	S_2_	S_3_	S_4_	S_5_	S_6_	S_7_	S_8_	S_9_	S_10_	S_11_	S_12_
S_1_	0	2	2	3	3	3	3	3	2	2	2	3
S_2_	3	0	3	3	2	3	3	3	3	3	3	3
S_3_	3	4	0	3	4	3	4	4	4	4	3	4
S_4_	3	3	3	0	3	4	3	3	3	3	3	3
S_5_	3	2	2	3	0	3	3	3	3	3	2	3
S_6_	1	3	1	2	2	0	2	1	2	2	1	1
S_7_	2	2	2	2	3	1	0	2	3	1	2	2
S_8_	3	3	2	2	2	1	2	0	3	2	2	2
S_9_	2	3	3	2	3	3	3	3	0	3	2	3
S_10_	3	3	3	3	3	3	4	4	3	0	4	3
S_11_	3	3	3	4	3	4	3	3	3	4	0	4
S_12_	3	3	2	3	3	3	3	3	3	3	3	0

#### Determination of the integrated impact matrix T

The combined impact matrix T is derived from Eqs. (1) to (2) and is shown in Table 3.

**Table 3:** Integrated impact matrix T.

	S_1_	S_2_	S_3_	S_4_	S_5_	S_6_	S_7_	S_8_	S_9_	S_10_	S_11_	S_12_
S_1_	0.17	0.229	0.201	0.245	0.251	0.25	0.261	0.256	0.235	0.222	0.206	0.249
S_2_	0.267	0.21	0.248	0.273	0.258	0.279	0.292	0.286	0.286	0.272	0.254	0.279
S_3_	0.313	0.349	0.219	0.319	0.349	0.325	0.365	0.357	0.358	0.34	0.297	0.348
S_4_	0.276	0.29	0.256	0.213	0.289	0.311	0.303	0.295	0.297	0.282	0.262	0.288
S_5_	0.252	0.242	0.213	0.257	0.194	0.263	0.276	0.27	0.27	0.256	0.218	0.262
S_6_	0.139	0.191	0.129	0.164	0.168	0.121	0.176	0.149	0.172	0.164	0.132	0.145
S_7_	0.187	0.195	0.174	0.191	0.218	0.173	0.157	0.201	0.223	0.169	0.177	0.196
S_8_	0.223	0.231	0.186	0.204	0.209	0.187	0.219	0.167	0.237	0.204	0.19	0.21
S_9_	0.232	0.266	0.236	0.237	0.266	0.264	0.278	0.272	0.203	0.259	0.22	0.264
S_10_	0.289	0.303	0.268	0.295	0.303	0.301	0.338	0.331	0.311	0.224	0.296	0.302
S_11_	0.295	0.31	0.274	0.324	0.31	0.331	0.324	0.317	0.317	0.323	0.212	0.33
S_12_	0.265	0.278	0.225	0.271	0.277	0.277	0.29	0.284	0.284	0.27	0.253	0.207

#### Determine relevant values and construct quadrant plots

**Figure 5:**
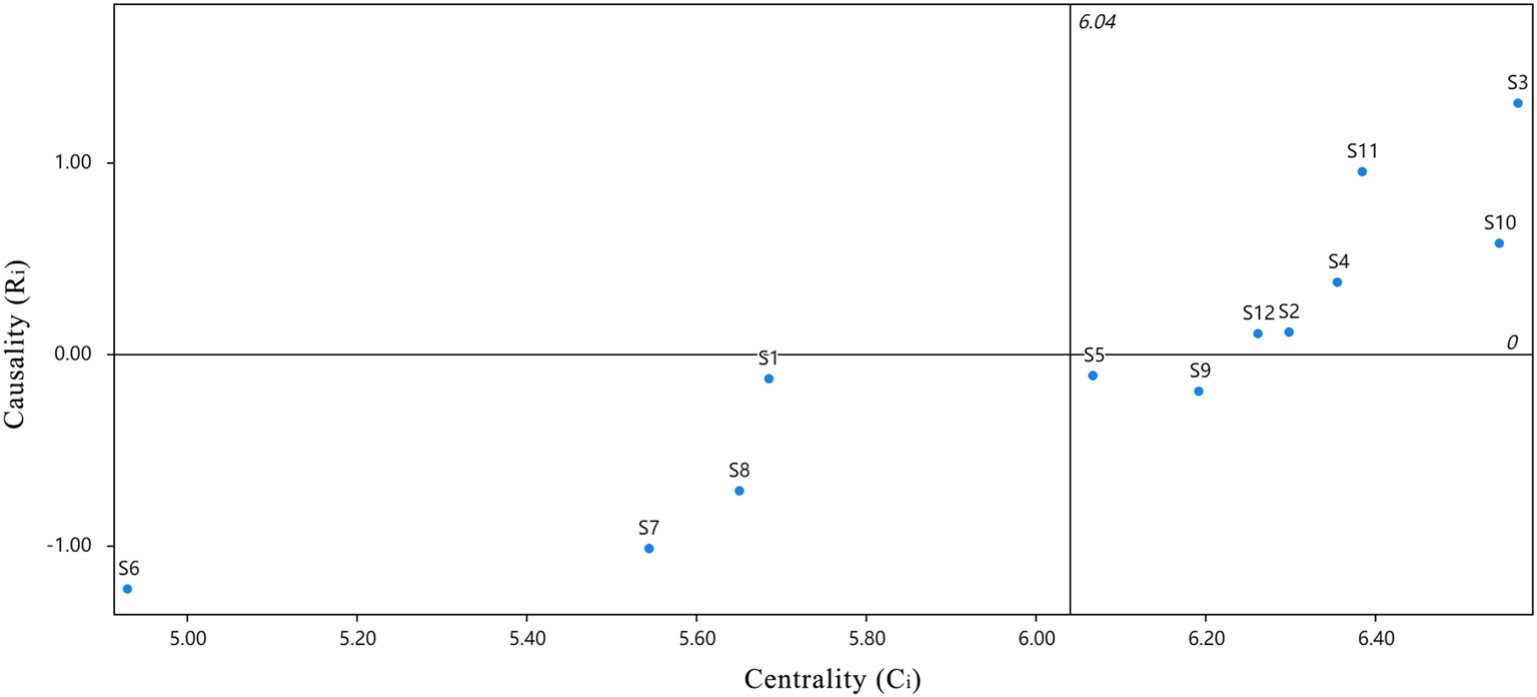
Degree of centrality and degree of cause diagram.

**Figure 6:**
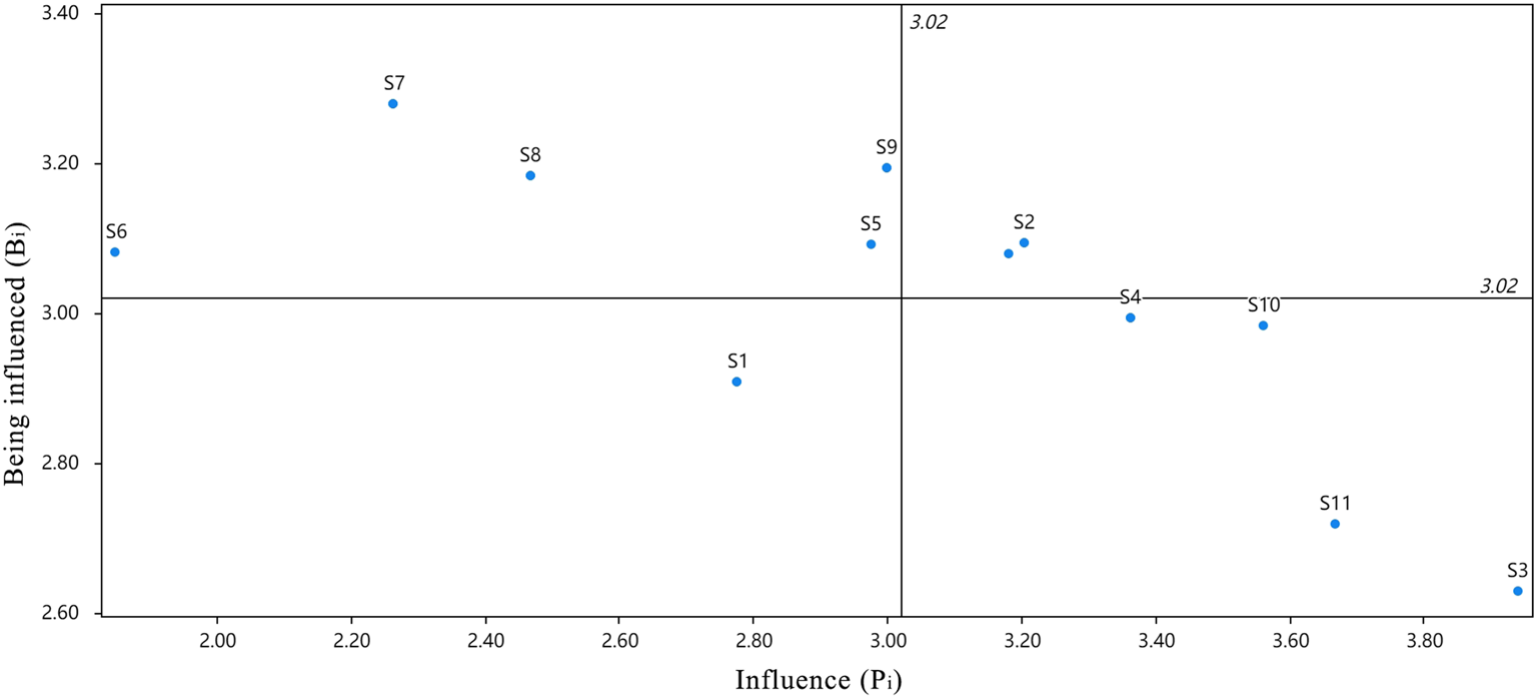
Influence and influenced graphs.

The four values were calculated according to Eqs. (3) to (6), respectively, and are shown in Table 4 and quadrant plots were created and are shown in Figs. 5 and 6.

**Table 4:** Combined effect relationships between factors.

	P_i_	B_i_	C_i_	R_i_	Weights	Arrange in order
S_1_	2.776	2.909	5.684	− 0.133	0.078	9
S_2_	3.203	3.095	6.298	0.109	0.087	5
S_3_	3.939	2.628	6.567	1.311	0.091	1
S_4_	3.362	2.993	6.355	0.369	0.088	3
S_5_	2.975	3.093	6.067	− 0.118	0.084	8
S_6_	1.849	3.081	4.93	− 1.233	0.068	12
S_7_	2.263	3.281	5.544	− 1.018	0.076	11
S_8_	2.467	3.183	5.65	− 0.717	0.078	9
S_9_	2.998	3.194	6.192	− 0.196	0.085	7
S_10_	3.561	2.984	6.545	0.576	0.09	2
S_11_	3.667	2.718	6.385	0.949	0.088	3
S_12_	3.181	3.08	6.261	0.101	0.086	6

### Calculations based on the ISM method

#### Calculation of the overall impact matrix

According to formula (7), the overall impact matrix F is calculated. Through computation, the threshold value $\gamma =0.252$ is obtained, and the reachable matrix Y is obtained, as shown in Table 5.

**Table 5:** Reachable matrix Y.

	S_1_	S_2_	S_3_	S_4_	S_5_	S_6_	S_7_	S_8_	S_9_	S_10_	S_11_	S_12_
S_1_	1	0	0	0	0	1	1	0	0	0	0	0
S_2_	0	1	0	0	1	1	1	1	1	0	0	0
S_3_	1	1	1	0	1	1	1	1	1	0	0	1
S_4_	0	0	0	1	1	1	1	1	1	0	0	0
S_5_	0	0	0	0	1	1	1	0	0	0	0	0
S_6_	0	0	0	0	0	1	0	0	0	0	0	0
S_7_	0	0	0	0	0	0	1	0	0	0	0	0
S_8_	0	0	0	0	0	1	0	1	0	0	0	0
S_9_	0	0	0	0	0	1	1	0	1	0	0	0
S_10_	1	1	0	0	1	1	1	1	1	1	0	1
S_11_	1	0	0	1	1	1	1	1	1	0	1	1
S_12_	1	0	0	0	1	1	1	1	0	0	0	1

#### Computational hierarchy models and relationship diagrams

**Figure 7:**
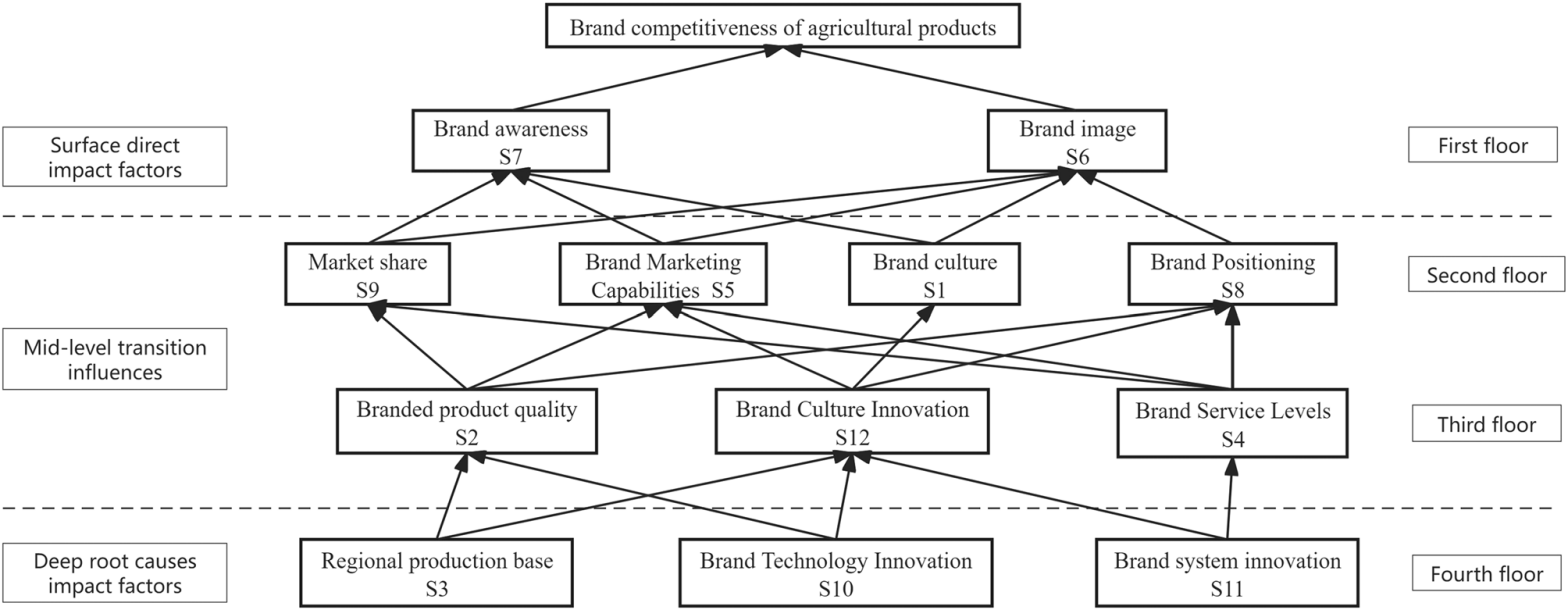
ISM Multi-level recursive structure of factors influencing brand competitiveness of agricultural products.

According to Eqs. (8) to (10), the hierarchical structure between the influencing factors is derived (as shown in Table 6), and a schematic diagram of the hierarchical relationship is derived based on the analysis of the relevant data, which is shown in Fig. 7.

**Table 6:** Segmentation of the set of influencing factors.

S_i_	Reachable set	Precursor series	Reachable set ∩ Precursor series
S_1_	S_1_, S_6_, S_7_	S_1_, S_3_, S_10_, S_11_, S_12_	S_1_
S_2_	S_2_, S_5_, S_6_, S_7_, S_8_, S_9_	S_2_, S_3_, S_10_	S_2_
S_3_	S_1_, S_2_, S_3_, S_5_, S_6_, S_7_, S_8_, S_9_, S_12_	S_3_	S_3_
S_4_	S_4_, S_5_, S_6_, S_7_, S_8_, S_9_	S_4_, S_11_	S_4_
S_5_	S_5_, S_6_, S_7_	S_2_, S_3_, S_4_, S_5_, S_10_, S_11_, S_12_	S_5_
S_6_	S_6_	S_1_, S_2_, S_3_, S_4_, S_5_, S_6_, S_8_, S_9_, S_10_, S_11_, S_12_	S_6_
S_7_	S_7_	S_1_, S_2_, S_3_, S_4_, S_5_, S_7_, S_9_, S_10_, S_11_, S_12_	S_7_
S_8_	S_6_, S_8_	S_2_, S_3_, S_4_, S_8_, S_10_, S_11_, S_12_	S_8_
S_9_	S_6_, S_7_, S_9_	S_2_, S_3_, S_4_, S_9_, S_10_, S_11_	S_9_
S_10_	S_1_, S_2_, S_5_, S_6_, S_7_, S_8_, S_9_, S_10_, S_12_	S_10_	S_10_
S_11_	S_1_, S_4_, S_5_, S_6_, S_7_, S_8_, S_9_, S_11_, S_12_	S_11_	S_11_
S_12_	S_1_, S_5_, S_6_, S_7_, S_8_, S_12_	S_3_, S_10_, S_11_, S_12_	S_12_

Based on the findings of hierarchical analysis and in conjunction with the associations among factors depicted in the adjacency matrix, we establish the ISM model for assessing the factors affecting agricultural product brand competitiveness. Illustrated in Fig. 7, the 12 influencing factors construct a hierarchical structure model with four tiers, demonstrating heterogeneous characteristics among factors across different levels. With the progression of tiers, the influencing factors of agricultural product brand competitiveness shift gradually from surface-level to deeper considerations.

## Conclusions and recommendations

### Conclusions

Enhancing agricultural economic growth benefits through branding to promote high-quality development within the agricultural economy is now a critical consideration in the emerging development paradigm. Viewing this issue from a national standpoint, agricultural product branding serves not only as an effective mechanism for expediting agricultural economic growth but also as a pivotal factor in guaranteeing the high quality and efficient supply of agricultural products, thereby contributing to the holistic advancement of the agricultural economy. Furthermore, significant disparities exist in the output value of agricultural products between the eastern and central-western regions, reflecting regional differences. Agricultural product output values tend to be higher in the eastern region but comparatively lower in the central-western region. Consequently, addressing this disparity necessitates regional adjustments to branding strategies tailored to capitalize on and accommodate the distinct conditions and advantages present in each region.

#### Analysis of surface-level direct influences.

In Fig. 7, the outermost layer comprises brand awareness (S_7_) and brand image (S_6_), constituting direct influencing factors of agricultural product brand competitiveness. Brands with high visibility in the agricultural sector typically engender trust among consumers, who are also more inclined to pay a premium for renowned brands. Consequently, agricultural product brands with high visibility can often command higher prices in the market, thus enhancing brand competitiveness^41^. A positive and consistent brand image also enhances consumer loyalty^29^. Within the agricultural product domain, continuously enhancing product quality, fostering brand awareness, and cultivating a positive brand image serve to bolster consumer perception and distinguish oneself in the market competition.

#### Analysis of factors influencing mid-level transitions

The seven factors situated at the intermediate level serve as "bridges" linking the surface-level direct influencing factors with the underlying influential factors. Through bridging the surface-level direct factors and the deep-rooted influencing factors, they facilitate transmission and transition. Notably, brand culture (S_1_), brand product quality (S_2_), brand service level (S_4_), and brand market positioning (S_8_) may directly or indirectly impact consumers' trust and attitudes towards the brand. Emotional connections between consumers and brands can be fostered from these angles, thereby cultivating a positive brand image and attracting more consumers to agricultural product brands. Furthermore, brand marketing capability (S_5_), market share (S_9_), and brand cultural innovation (S_12_) impact the brand competitiveness of agricultural products from internal and external standpoints. Hence, agricultural product brands ought to stay abreast of contemporary trends, guiding consumers towards emerging demands for agricultural products while bolstering the brand's foresight. Moreover, strategic market promotion and active engagement in advertising endeavors can elevate brand awareness and recognition, consequently augmenting market share.

#### Deep root cause impact factor analysis

In Fig. 7, the foundational factors for agricultural product brand competitiveness are delineated as regional production foundation (S_3_), brand technological innovation (S_10_), and brand institutional innovation (S_11_). These factors represent the fundamental influences on brand competitiveness. Certain region-specific attributes like soil quality and climate can give rise to geographical indications, facilitating traceability and quality assurance for agricultural product brands. This phenomenon not only boosts brand credibility but also serves as distinctive selling points for the brand^42^. Furthermore, the integration of novel technologies contributes to enhancing the quality of agricultural products. This includes augmenting yields, minimizing losses, and refining processing methodologies, consequently bolstering the competitiveness of agricultural products^43^. Moreover, the optimization of the industry chain necessitates collaborative efforts spanning production, processing, logistics, and other related sectors. Consequently, agricultural product brands can achieve cost reductions and efficiency enhancements.

### Recommendations

Investigating the competitive landscape of agricultural product brands stands as a pivotal endeavor within the realm of agricultural advancement. Using agricultural products from the Yangtze River Delta region of China as an illustrative case study, this study provides a comprehensive summary and analysis of the factors influencing the competitiveness of agricultural product brands. This study constructs a hierarchical influence pathway model to elucidate the interrelationships among the factors influencing the competitiveness of agricultural product brands. With regard to the underlying influential factors depicted in Fig. 7, suggestions and strategies for enhancing the competitiveness of agricultural product brands are put forward, focusing on three dimensions: regional production base (S_3_), brand technological innovation (S_10_), and brand institutional innovation (S_11_).

#### Enhance the production infrastructure and rationally establish developmental objectives

Enhancing the competitiveness of agricultural product brands from the perspective of regional production bases necessitates the full exploitation of regional characteristics and advantages. Initially, exploring the region's climate, soil, and water resources is crucial to cultivating agricultural products that embody local distinctiveness, thereby establishing unique brand selling points. Additionally, securing geographical indication certification solidifies the association of agricultural products with their specific regions, which significantly boosts their traceability and credibility.

Secondly, to enhance the regional distribution of agricultural products in the Yangtze River Delta and mitigate the issue of homogeneity in market competition, it is crucial to pursue misaligned development strategies and encourage differentiated competition. Establishing robust standards for agricultural product quality and implementing a strategic program to improve varieties based on regional resource endowments are essential. This strategy should include national planning for the layout of variety improvements. Additionally, support the creation of national, regional, and local zones for agricultural product brand innovation to ensure the stability and reliability of product quality. Moreover, increased investment in rural infrastructure is necessary to improve production conditions and the efficiency of agricultural product distribution^44^.

Ultimately, fostering the integration of agriculture with other sectors is essential to developing a diversified economic model and increasing the added value of the agricultural supply chain. Concurrently, it is crucial to develop mechanisms for the dissemination and sharing of scientific findings and to enhance collaborations among universities, research institutions, and businesses focused on scientific exploration and talent cultivation. A collaborative framework should be established, involving governmental intellectual property protection agencies, industry associations, cooperatives, and agricultural enterprises, to support a multifaceted protection system. Embracing contemporary concepts of market supervision, innovating regulatory frameworks, and nurturing organizations that provide social services for agricultural product brands are imperative for advancing this agenda^45^.

#### Lead technological innovation and form a synergistic force for reform and innovation

From the standpoint of brand technological innovation, considering the high homogeneity and low differentiation typical of agricultural products, the primary objective in their management is to innovate in cultivation and breeding techniques. Such innovation is essential for achieving product differentiation, thereby allowing marketed products to attain high added value attributed to their 'scarcity' and 'innovation'.

Firstly, given the inherent seasonal nature of agricultural products, adopting both basic and advanced processing strategies emerges as an effective avenue for corporate innovation^46^. Investing in the processing of agricultural products—such as dried fruits, canned goods, and dehydrated fruits—and further innovating with reprocessed items like dates stuffed with walnuts, sesame-filled seaweed, and filled persimmon cakes, not only mitigates the seasonal supply gaps but also opens new avenues for product innovation within companies. Moreover, leveraging genetic modification technologies to develop new varieties with enhanced yields and improved pest and disease resistance reduces dependence on chemical pesticides, thereby enhancing product safety and environmental sustainability, which in turn boosts the market competitiveness of these companies.

Secondly, to boost the global competitiveness of agriculture, it is essential to strategically leverage both domestic and international markets and resources. This includes taking a leadership role in establishing relevant international standards and rules, developing world-renowned agricultural product brands, and promoting the trade of unique and advantageous agricultural products to further their internationalization^47^. Additionally, utilizing e-commerce platforms for the digital marketing of agricultural products not only enhances product visibility and expands sales channels but also employs advanced technologies like blockchain to increase supply chain transparency. This approach ensures product traceability, thereby building consumer trust—a crucial factor in enhancing the market competitiveness of agricultural products.

Finally, to effectively advance the integration of agricultural product distribution, developing a strategic spatial layout for agricultural product circulation and removing inter-regional distribution barriers are essential. This strategy involves increasing investment in transportation infrastructure and equipment for agricultural products, enhancing the control capabilities of key logistics nodes, and ensuring seamless agricultural production, distribution, and consumption processes. Furthermore, it is imperative to accelerate the enhancement of informatization in transportation infrastructure, develop multimodal transport operators, and bolster the construction of warehousing, preservation, and cold chain logistics facilities at agricultural production sites to foster the rapid development of rural e-commerce^48^. Additionally, policies should encourage modern distribution enterprises to focus more on the central and western regions, optimize and upgrade internal commercial and logistics networks, and integrate global resources to establish cost-effective, efficient, and highly resilient distribution channels. Exploring a 'Smart+' model for the intelligent and informatized development of agriculture will facilitate the integration of agricultural products from the central and western regions into the new dual-circulation development paradigm, both domestically and internationally.

#### Promote institutional upgrades to support the healthy development of brands

From the perspective of brand system innovation, agricultural enterprises exert a pivotal guiding role in the market. Firstly, regional policies must focus on enhancing the protection and management of agricultural product brands, particularly by securing certifications based on geographical indications. Such certifications underscore the vital contribution of local specialty agricultural products to rural industrial development, improvement of the primary industry's quality and efficiency, and augmentation of farmers' incomes. Additionally, the benefits of large-scale agricultural operations should be leveraged to optimize and expand the industry and value chains of agricultural products, thereby increasing the brands' added value and leading effectively in market competition and demonstration^49^. Furthermore, a robust collaborative mechanism within the agricultural industry chain should be established to motivate producers to form cooperatives and alliances. This approach fosters close collaboration throughout the agricultural industry chain and facilitates the sharing of resources, information, and market opportunities.

To safeguard the safety of agricultural products, it is advisable to establish a comprehensive safety production system and implement full-process risk management spanning from production to sales, facilitated by digital management systems. This initiative will bolster control capabilities over agricultural product safety, thereby ensuring consumers' access to safe and high-quality agricultural products. Regarding marketing and brand maintenance, enhancing synergies between government entities and market participants is imperative. Specifically, government agencies should selectively support leading enterprises within the agricultural products sector, augmenting their financial, technological, and personnel support to foster innovation and development in the agricultural products market^50^. Moreover, the government should steer agricultural enterprises towards transitioning from traditional offline marketing models to online + offline omnichannel marketing strategies, aligning with the purchasing preferences of contemporary consumers and broadening market reach.

In conclusion, certain local governments and enterprises exhibit inadequate marketing awareness and deficient brand communication capabilities, leading to their brand influence being confined to the local realm. To augment the impact of agricultural product brands, local governments should intensify their focus on brand communication and implement a series of measures to bolster enterprise brand marketing endeavors^51^. For instance, local authorities should proactively facilitate collaborations between media, marketing, e-commerce, and relevant university departments to establish university-enterprise alliances with local agricultural product brand enterprises, thereby furnishing enterprises with high-quality brand communication support. Moreover, local governments can devise incentive policies to recognize enterprises and individuals who have made noteworthy contributions to agricultural industry chain innovation, thereby fostering further institutional innovation and motivating enterprises.

## Discussion and limitations

### Significance of the study

From an academic standpoint, this research comprehensively examines the developmental mechanisms and influencing factors of agricultural product brands in the Yangtze River Delta region, elucidates the interplay between brand construction and market competition, and furnishes a theoretical framework for comprehending the operational dynamics of the agricultural product market. Furthermore, delving into the competitiveness of agricultural product brands in the Yangtze River Delta region offers theoretical underpinning for advancing agricultural modernization and rural revitalization. Moreover, delving into the competitiveness of agricultural product brands can generate abundant research avenues and theoretical insights across various disciplines such as economics, marketing, and agricultural economics, thereby offering guidance for regional economic development models, adjustments in industrial structure, and related matters.

Examining the competitiveness of agricultural product brands is crucial for guiding the advancement of the regional agricultural product industry. Comprehending the present competitive landscape and developmental trends of agricultural product markets in the Yangtze River Delta can inform strategic brand positioning and marketing for agricultural enterprises, thereby boosting their market competitiveness and profitability. Additionally, examining the competitiveness of agricultural product brands in the Yangtze River Delta region can serve as a model for developing agricultural product brands in other areas, thus fostering nationwide improvement and elevation of agricultural product brands. Moreover, bolstering the establishment of agricultural product brands in the Yangtze River Delta region aids in augmenting the visibility and reputation of regional agricultural products, facilitating both domestic and international sales, and facilitating the effective realization of strategies for rural economic sustainability and revitalization.

## Research limitations

This study examines the determinants influencing the competitiveness of agricultural product brands. However, several deficiencies persist. Primarily, the study centers on agricultural product brands within the Yangtze River Delta region, encompassing provinces exhibiting varied economic development and agricultural production profiles, including Shanghai, Jiangsu, Zhejiang, and Anhui. Substantial disparities exist in the developmental status of agricultural product brands and market conditions across different regions. Regrettably, these variations remain unexplored in the study, constituting a significant limitation of the research design. Secondly, the analysis solely examines the influencing factors of agricultural product brands, overlooking their role in regional economic development, social dynamics, transportation infrastructure, and other relevant aspects. Lastly, China is presently transitioning its rural revitalization strategy from macro policy formulation to micro-level implementation. Agricultural product brands emerge as potent instruments in this process. Nevertheless, the empirical investigation into the efficacy of agricultural brands during this transitional phase remains inadequately comprehensive. Government policies significantly shape the trajectory of agricultural product brand development. Given the potential evolution of policy environments and support levels in the Yangtze River Delta region, there exists uncertainty that could impinge upon the long-term applicability and generalizability of the research findings.

Future research could investigate how different provinces and cities in the Yangtze River Delta can collaborate to improve the overall competitiveness of agricultural product brands. This involves examining resource sharing, policy coordination, and market integration strategies among diverse regions, and understanding their impacts on brand development and market competitiveness. Additionally, future research should account for the variations and distinguishing features of agricultural product brands across provinces within the Yangtze River Delta region. Researchers could choose specific agricultural product brands for field studies and conduct thorough research on their contributions to increasing farmers' income and fostering industrial development. Lastly, given the accelerated pace of globalization, future research should examine the adaptation of Yangtze River Delta agricultural products to international market demands, and explore methods to enhance brand competitiveness globally through international cooperation and external market strategies.

## Data Availability

The datasets used and analysed during the current study available from the corresponding author on reasonable request.

## References

[1] Long, Y. Export competitiveness of agricultural products and agricultural sustainability in China. *Reg. Sustain.* **2**, 203–210 (2021).

[2] Liu, X. Structural changes and economic growth in China over the past 40 years of reform and opening-up. *China Polit. Econ.* **3**, 19–38 (2020).

[3] Reardon, T. & Timmer, C. P. Chapter 55: Transformation of markets for agricultural output in developing countries since 1950: How has thinking changed? In *Handbook of Agricultural Economics* Vol. 3 (eds Evenson, R. & Pingali, P.) 2807–2855 (Elsevier, New York, 2007).

[4] Wang, Y. Discussion on the regional brand development of agricultural products. *IOP Conf. Ser. Earth Environ. Sci.* **598**, 012055 (2020).

[5] Zhang, Z. The, “three rural” issues in China’s market-oriented economic reform. In *Handbook of Chinese Economics* (ed. Zhang, Z.) 391–477 (Springer Nature, 2023). 10.1007/978-981-99-0420-4_14.

[6] Wang, S., Yuan, L. & Gong, B. China’s agricultural green transition and high-quality development toward carbon neutrality. *Chin. Polit. Sci. Rev.* **8**, 240–272 (2023).

[7] Wei, H., Tan, X., Gao, L., Zhang, R. & Cui, K. Research on rural vitalization and anti-poverty strategy and policy. In *The New Journey of China’s Economic and Social Development* (eds Xie, F. *et al.*) 129–178 (Springer, 2023). 10.1007/978-981-19-7915-6_6.

[8] Li, X. Development of agricultural product logistics in China. In *Contemporary Logistics in China: Systemic Reconfiguration and Technological Progression* (eds Jiao, Z. *et al.*) 145–176 (Springer, 2021). 10.1007/978-981-16-5580-7_7.

[9] Yang, J., Chang, J., Konar, M., Wang, Y. & Yao, J. The grain Food-Energy-Water nexus in China: Benchmarking sustainability with generalized data envelopment analysis. *Sci. Total Environ.* **887**, 164128 (2023). 37172834 10.1016/j.scitotenv.2023.164128

[10] Hu, D., Reardon, T., Rozelle, S., Timmer, P. & Wang, H. The emergence of Supermarkets with Chinese characteristics: Challenges and opportunities for China’s Agricultural Development. *Development Policy Review* **22**, 557–586 (2004).

[11] Zhou, Y., Li, X. & Liu, Y. Cultivated land protection and rational use in China. *Land Use Policy* **106**, 105454 (2021).

[12] Fan, Y., Fang, C. & Zhang, Q. Coupling coordinated development between social economy and ecological environment in Chinese provincial capital cities-assessment and policy implications. *J. Clean. Prod.* **229**, 289–298 (2019).

[13] Huang, J., Otsuka, K. & Rozelle, S. Agriculture in China’s development: Past Disappointments, recent successes, and future challenges. *China’s Great Economic Transformation*10.1017/cbo9780511754234.014 (2008).

[14] Sanders, R. A market road to sustainable agriculture? Ecological Agriculture, green food and organic agriculture in China. *Dev. Chang.* **37**, 201–226 (2006).

[15] Wang, Y. *et al.* The interaction relationships among agricultural certification labels or brands: Evidence from Chinese consumer preference for fresh produce. *Int. Food Agribus. Manag. Rev.*10.22434/IFAMR2021.0048 (2021).

[16] Xiao, J., Wang, W. & Tsai, S.-B. Coupling of agricultural product marketing and agricultural economic development based on big data analysis and “Internet+”. *Mob. Inf. Syst.* **2021**, e3702064 (2021).

[17] Xu, D., Lin, Y., Li, C. & Tong, T. Research on the path to brand-building of characteristic agricultural products. In *Simulation Tools and Techniques* (eds Song, H. & Jiang, D.) 495–512 (Springer International Publishing, 2021). 10.1007/978-3-030-72792-5_40.

[18] Xu, M. Exploration of strategies for improving brand competitiveness of agricultural products in the context of digitization. *Appl. Math. Nonlinear Sci.* (2023).

[19] Galati, A., Tulone, A., Tinervia, S. & Crescimanno, M. The role of internal resources in the competitive positioning of Sicilian wine cooperatives. *Int. J. Global. Small Bus.* **10**, 324–337 (2019).

[20] Abimbola, T. Branding as a competitive strategy for demand management in SMEs. *J. Res. Mark. Entrep.* **3**, 97–106 (2001).

[21] Sgroi, F. Digital technologies to remove the information asymmetry in the food market. *Smart Agricult. Technol.* **5**, 100326 (2023).

[22] Jambor, A. & Babu, S. The competitiveness of global agriculture. In *Competitiveness of Global Agriculture,* 99–129 (Springer, 2016). 10.1007/978-3-319-44876-3_6.

[23] Gardner, B. B. The product and the brand. *Harvard Bus. Rev.* **33**, 33 (1955).

[24] Motameni, R. & Shahrokhi, M. Brand equity valuation: a global perspective. *J. Prod. Brand Manag.* **7**, 275–290 (1998).

[25] Cai, T., Dai, H.-C. & Song, H.-X. Research on the evaluation model of brand competitiveness of power enterprises based on the fuzzy comprehensive evaluation method. In *Fuzzy System and Data Mining* 17–23 (IOS Press, 2016). 10.3233/978-1-61499-619-4-17.

[26] Gupta, S., Gallear, D., Rudd, J. & Foroudi, P. The impact of brand value on brand competitiveness. *J. Bus. Res.* **112**, 210–222 (2020).

[27] Liu, Y. & Wang, X. Promoting competitiveness of green brand of agricultural products based on agricultural industry cluster. *Wirel. Commun. Mob. Comput.* **2022**, e7824638 (2022).

[28] Jin, Y. & Du, Y. Analysis on the Factors Influencing the International Competitiveness of Shanxi Agricultural Products, 168–175 (Atlantis Press, 2020). 10.2991/aebmr.k.201211.031.

[29] Anselmsson, J., Vestman Bondesson, N. & Johansson, U. Brand image and customers’ willingness to pay a price premium for food brands. *Journal of Product Brand Management* **23**, 90–102 (2014).

[30] Tao, M. & Li, Z. Research on the construction of enterprise brand competitiveness evaluation system based on the integration of SWOT and AHP Model. In *Recent Developments in Data Science and Business Analytics* (eds Tavana, M. & Patnaik, S.) 55–62 (Springer International Publishing, 2018). 10.1007/978-3-319-72745-5_6.

[31] Frohberg, K. & Hartmann, M. *Comparing Measures of Competitiveness*. https://www.econstor.eu/handle/10419/28566 (1997).

[32] Fernandes, J. & Nogueira, S. P. Cooperation as a key factor to increase local companies’ competitiveness: a descriptive analysis. (IBIMA Publishing, 2019).

[33] Li, N., Song, Y., Xia, W. & Fu, S.-N. Regional transportation integration and high-quality economic development, coupling coordination analysis, in the Yangtze River Delta, China. *Systems* **11**, 279 (2023).

[34] Cao, J. *et al.* Public awareness of remanufactured products in Yangtze River Delta of China: Present status, problems and recommendations. *Int. J. Environ. Res. Public Health* **15**, 1199 (2018). 29880752 10.3390/ijerph15061199 PMC6024935

[35] Al-Naggar, A. M. Emerging issues in agricultural. *Sciences*10.9734/bpi/eias/v6 (2023).

[36] Yan, D. & Sun, W. Study on the evolution, driving factors, and regional comparison of innovation patterns in the Yangtze River Delta. *Land* **11**, 876 (2022).

[37] Yang, J. *et al.* Current situation, bottlenecks, and path options for the development of capital flows and integration in the Yangtze River Delta region. *Front. Sustain. Cities* **5** (2023).

[38] Northcutt, N. & McCoy, D. *Interactive Qualitative Analysis: A Systems Method for Qualitative Research*. xxvii, 441 (Sage Publications, Inc, 2004). 10.4135/9781412984539.

[39] Seyed-Hosseini, S. M., Safaei, N. & Asgharpour, M. J. Reprioritization of failures in a system failure mode and effects analysis by decision making trial and evaluation laboratory technique. *Reliab. Eng. Syst. Saf.* **91**, 872–881 (2006).

[40] Watson, R. H. Interpretive structural modeling—A useful tool for technology assessment?. *Technol. Forecast. Soc. Change* **11**, 165–185 (1978).

[41] Porter, M. E. Industry structure and competitive strategy: Keys to profitability. *Financ. Anal. J.*10.2469/faj.v36.n4.30 (1980).

[42] Kontogeorgos, A. Brands, quality badges and agricultural cooperatives: how can they co-exist?. *TQM J.* **24**, 72–82 (2012).

[43] Benyam, A. (Addis), Soma, T. & Fraser, E. Digital agricultural technologies for food loss and waste prevention and reduction: Global trends, adoption opportunities and barriers. *J. Cleaner Prod.* **323**, 129099 (2021).

[44] Zu, X., He, Y., Pu, Y. & Yang, L. The characteristics of agricultural product quality and safety crisis based on content analysis method. In *Proceedings of the Fourteenth International Conference on Management Science and Engineering Management* (eds Xu, J. *et al.*) 119–129 (Springer International Publishing, 2020). 10.1007/978-3-030-49829-0_9.

[45] Wang, Y. & Huan, M. The effects of socialized agricultural services on rural collective action in the irrigation commons: Evidence from China. *Agricultural Water Management* **289**, 108519 (2023).

[46] Adenle, A. A., Manning, L. & Azadi, H. Agribusiness innovation: A pathway to sustainable economic growth in Africa. *Trends Food Sci. Technol.* **59**, 88–104 (2017).

[47] Zhou, L. & Tong, G. Research on the competitiveness and influencing factors of agricultural products trade between China and the countries along the “Belt and Road”. *Alex. Eng. J.* **61**, 8919–8931 (2022).

[48] Han, J.-W. *et al.* A comprehensive review of cold chain logistics for fresh agricultural products: Current status, challenges, and future trends. *Trends Food Sci. Technol.* **109**, 536–551 (2021).

[49] Zhai, T., Liu, J. & Wang, D. Optimization path of agricultural products marketing channel based on innovative industrial chain. *Econ Change Restruct* **56**, 3949–3977 (2023).

[50] Zhang, W., Huang, M., Shen, P. & Liu, X. Can digital inclusive finance promote agricultural green development?. *Environ. Sci. Pollut. Res.*10.1007/s11356-023-29557-8 (2023). 10.1007/s11356-023-29557-8 37691061

[51] Yao, Q., Gong, S. & Wei, H. Marketing capabilities drive competitive advantages: Evidence from China’s agribusinesses. *Agric. Res.* **5**, 305–315 (2016).

